# P-844. Bacteraemia Caused by High Risk AmpC-producing Enterobacterales: Influence of IDSA Guidelines Adequacy and Antimicrobial Stewardship Activity on Outcomes

**DOI:** 10.1093/ofid/ofae631.1036

**Published:** 2025-01-29

**Authors:** Jose Luis Lamas Ferreiro, Ana Sanjurjo Rivo, Judith Alvarez Ortero, Alejandra Canoa Rico, Marta Costas Vila, Monica Gutierrez Garcia, Laura Intanno Valerio, Javier de la Fuente Aguado

**Affiliations:** Ribera POVISA Hospital, Vigo, Galicia, Spain; Hospital Povisa, Vigo, Galicia, Spain; Mayo Clinic, Rochester, Minnesota; Ribera Povisa Hospital, Vigo, Galicia, Spain; Hospital Povisa, Vigo, Galicia, Spain; Ribera Povisa Hospital, Vigo, Galicia, Spain; Ribera Povisa Hospital, Vigo, Galicia, Spain; Hospital Povisa, Vigo, Galicia, Spain

## Abstract

**Background:**

Treatment of high risk AmpC-producing Enterobacterales bloodstream infections (BSIs) is challenging due to the posibility of development of antibiotic resistance during treatment. The aim of this study was to analyze the impact of antimicrobial stewardship activity and IDSA guidelines adequacy in patients with high risk AmpC-producing Enterobacterales BSIs.

**Methods:**

Patients with *Citrobacter* spp, *Enterobacter* spp and *Klebsiella aerogenes* BSIs were included between June 2019-March 2024. Outcomes of death and relapse of infection at 30 days were assessed. Adequacy of definitive antimicrobial treatment according to IDSA guidelines was evaluated. The impact of antimicrobial stewardship activity in treatment adequacy and outcome was analyzed. Analysis was performed using SPSS version 25.

**Results:**

2049 BSIs were evaluated and 67 were finally included. 80% were monomicrobial. Median patient age was 74 years, 73% were male. The most frequent microorganism isolated was *Enterobacter cloacae* (55%). 50% were nosocomial and the most frequent origen was biliary tract (35,8%). 35% met sepsis criteria. 30% of cases presented cefotaxim resistance. Empiric and definitive antibiotic treatment was prescribed according IDSA guidelinesi n 34% and 91% of cases, respectively.. Death at 30 days was 6% and a combination outcome of death and relapse of infection at 30 days was 11,9%. Definitive antibiotic treatment adequacy according to IDSA guidelines was associated with lower mortality and relapse at 30 days (OR 0,04; IC95 0,003-0,6; P=0,02). Antimicrobial stewardship team did an intervention in 34% cases. This intervention was associated with a trend toward of lower mortality and relapse (4% vs 15%, P=0,2), a trend toward of higher IDSA guidelines adequacy (100% vs 86%, P=0,08), and a higher switch to oral treatment (78% vs 52%; P=0,03).

**Conclusion:**

Adequacy of definitive antimicrobial treatment in high risk AmpC producing Enterobacterales BSIs according to IDSA guidelines was associated with a better outcome. Antimicrobial stewardship is desired for management of these patients.

**Disclosures:**

**All Authors**: No reported disclosures

